# The influence of family on health anxiety in frequently ill adolescents

**DOI:** 10.1192/j.eurpsy.2022.1079

**Published:** 2022-09-01

**Authors:** I. Shishkova, E. Pervichko

**Affiliations:** 1 Ryazan State Medical University named after I.P. Pavlov, Faculty Of Clinical Psychology, Ryazan, Russian Federation; 2 Lomonosov Moscow State University, Faculty Of Psychology, Moscow, Russian Federation; 3 Pirogov Russian National Research Medical University, Clinical Psychology Department, Moscow, Russian Federation

**Keywords:** health, subjective pattern of health, health anxiety, frequently ill adolescents

## Abstract

**Introduction:**

Adolescents (especially frequently ill) from families where parents show high concern for their health, often themselves make complaints about their health status that do not receive medical confirmation (Kovalenko, 1998; Dielman et al., 1991). A study by T. Dillman and colleagues (1991) revealed a direct link between the perception of the disease in parents and children – the more seriously the parent perceives the child’s condition, the more seriously the child treats it, and the more complaints he has.

**Objectives:**

To study the influence of family on health anxiety in frequently ill adolescents.

**Methods:**

The sample: 98 adolescents (mean age 16.1±0.9), 84 their parents (mean age 44.5±5.0). We used: “Short Health Anxiety Inventory” (SHAI; Salkovskis et al., 2002), The “Research on health-saving activities” (RHSA) questionnaire (Yakovleva, 2014), Questionnaire “Index of attitude toward health” (Deryabo, Yasvin, 1999).

**Results:**

The results of multiple regression analysis showed that health anxiety in adolescents is determined by the following parent’s features: goal-setting in the field of health-preserving activity (-0.661, p=0.036), standards of health (0.518, p=0.028), self-efficacy in the field of health-preserving activity (0.892, p=0.010), cognitive scale of attitude toward health (0.586, p=0.032) and scale of actions (0.059, p=0.002). It is also determined by parents’ vigilance to bodily sensations (0.815, p=0.000).

**Conclusions:**

Health anxiety in adolescents is influenced by both cognitive, motivational and behavioral components of the attitude toward health of their parents, and also sensory (negative physical sensations and symptoms in parents form anxiety about health of their children). Research is supported by the Russian Science Foundation, project No. 21-18-00624.

**Disclosure:**

Research is supported by the Russian Science Foundation, project No. 21-18-00624.

